# Interobserver Agreement Rates on CXCR4-Directed PET/CT in Patients with Marginal Zone Lymphoma

**DOI:** 10.1007/s11307-024-01940-y

**Published:** 2024-08-01

**Authors:** Rudolf A. Werner, Yingjun Zhi, Niklas Dreher, Samuel Samnick, Aleksander Kosmala, Takahiro Higuchi, Lena Bundschuh, Constantin Lapa, Andreas K. Buck, Max S. Topp, Hermann Einsele, Johannes Duell, Sebastian E. Serfling, Ralph A. Bundschuh

**Affiliations:** 1 Department of Nuclear Medicine, Clinic for Radiology and Nuclear Medicine, Goethe University, University Hospital Frankfurt, Frankfurt, Germany; 2 Department of Radiology and Radiological Science, The Russell H. Morgan, Johns Hopkins University School of Medicine, Baltimore, MD USA; 3 Department of Otorhinolaryngology, Plastic, Aesthetic and Reconstructive Head and Neck Surgery, University Hospital Würzburg, Würzburg, Germany; 4 Department of Nuclear Medicine, University Hospital of Würzburg, Würzburg, Germany; 5 Graduate School of Medicine, Dentistry and Pharmaceutical Sciences, Okayama University, Okayama, Japan; 6 Nuclear Medicine, Medical Faculty, University of Augsburg, Augsburg, Germany; 7 Medical Department II, University Hospital Würzburg, Würzburg, Germany

**Keywords:** Marginal zone lymphoma, CXCR4, C-X-C motif chemokine receptor 4, Chemokine receptor, Theranostics

## Abstract

**Abstract:**

C-X-C motif chemokine receptor 4 (CXCR4)-directed molecular imaging provides excellent read-out capabilities in patients with marginal zone lymphoma (MZL). We aimed to determine the interobserver agreement rate of CXCR4-targeted PET/CT among readers with different levels of experience.

**Methods:**

50 subjects with MZL underwent CXCR4-targeted PET/CT, which were reviewed by four readers (including two experienced and two less experienced observers). The following 8 parameters were investigated: overall scan result, CXCR4 density in lymphoma tissue, extranodal organ involvement, No. of affected extranodal organs and extranodal organ metastases, lymph node (LN) involvement and No. of affected LN areas and LN metastases. We applied intraclass correlation coefficients (ICC; < 0.4, poor; 0.4–0.59, fair; 0.6–0.74, good and > 0.74 excellent agreement rates).

**Results:**

Among all readers, fair agreement was recorded for No. of affected extranodal organs (ICC, 0.40; 95% confidence interval [CI], 0.25–0.68), overall scan result (ICC, 0.42; 95%CI, 0.28–0.57), CXCR4 density in lymphoma tissue (ICC, 0.52; 95%CI, 0.38–0.66), and No. of extranodal organ metastases (ICC, 0.55; 95%CI, 0.41–0.61) and LN involvement (ICC, 0.59; 95%CI, 0.46–0.71). Good agreement rates were observed for No. of LN metastases (ICC, 0.71; 95%CI, 0.60–0.81) and No. of LN areas (ICC, 0.73; 95%CI, 0.63–0.82), while extranodal organ involvement (ICC, 0.35; 95%CI, 0.21–0.51) achieved poor concordance. On a reader-by-reader comparison, the experienced readers achieved significantly higher agreement rates in 4/8 (50%) investigated scan items (ICC, range, 0.21–0.90, P < / = 0.04). In the remaining 4/8 (50%), a similar trend with higher ICCs for the experienced readers was recorded (n.s.).

**Conclusion:**

CXCR4-directed PET/CT mainly provided fair to good agreement rates for scan assessment, while a relevant level of experience seems to be required for an accurate imaging read-out.

**Supplementary Information:**

The online version contains supplementary material available at 10.1007/s11307-024-01940-y.

## Introduction

Marginal zone lymphoma (MZL) is characterized by an intense expression of the C-X-C motif chemokine receptor 4 (CXCR4) in sites of disease [1]. Thus, this lymphoma subtype has been extensively evaluated using the theranostics CXCR4 PET probe [^68^ Ga]Ga-PentixaFor to report on the current status of chemokine receptor expression (2–6). Applying PET-piloted biopsies, Duell et al. were the first to demonstrate a relevant association between [^68^ Ga]Ga-PentixaFor PET signal and ex-vivo chemokine receptor upregulation [7]. In this preliminary report investigating 20 patients with MZL, chemokine receptor imaging identified a relevant rate of upstaged individuals, which also triggered treatment changes [7]. Those initial findings have been recently further corroborated in a cohort of 100 subjects affected with MZL [2]. Comparing [^68^ Ga]Ga-PentixaFor with CT and other guideline-compatible diagnostic tools in the work-up of MZL, molecular imaging caused stage migration based on Ann Arbor (AA) classification in 27%, mainly by re-classifying patients to the clinically relevant category of AA III or IV [2]. As MZL also presents with gastral involvement, chemokine receptor PET also provided an accuracy between 76 to 94% relative to esophagogastroduodenoscopy and bone marrow biopsy [2]. In those patients experiencing staging changes, molecular imaging then caused different hemato-oncological therapeutic algorithms in more than 85% [2]. Beyond diagnostic impact, approximately one fifth of the patients would also have been suitable for radioligand therapy (RLT) using the therapeutic ß-emitting [^177^Lu]Lu/[^90^Y]Y-PentixaTher [2]. As such, those promising results may favor a more routine use of [^68^ Ga]Ga-PentixaFor in MZL, which may even obviate the need of strenuous procedures such as biopsies. Nonetheless, prior to a more widespread adoption, clinicians and nuclear medicine physicians should rest assured that imaging results provide a high concordance among multiple readers [8–10], in particular for Gallium-68 labeled PET agents associated with less favorable physical and chemical properties when compared to fluorinated agents [11]. In this regard, a recent study reported on at least fair concordance rates for solid cancers imaged with [^68^ Ga]Ga-PentixaFor [12]. Given the relevant impact of this PET probe for MZL [2, 7], we aimed to define its interobserver agreement rate for MZL patients among readers with different experience levels. We focused on varying diagnostic scan parameters.

## Material and Methods

We investigated 50 subjects affected with MZL in this retrospective analysis and imaged those individuals with [^68^ Ga]Ga-PentixaFor PET/CT (after written informed consent). 45/50 (90%) were referred to our imaging center for staging (5/50 [10%] for restaging). Most patients (27/50 [54%]) were affected with nodal disease (Table 1). Local ethics committee waived the need for approval (#20,210,726 02). This cohort has been partially reported in [2, 3, 5, 7], but without investigating interobserver agreement rates among multiple readers with different levels of experience focusing on diagnostic parameters.

**Table 1:** Patient’s characteristics. Percentages are given in brackets. *Mean ± standard deviation

Male		18/50 (36)
Age		64.6 ± 20.7*
Scan indication	Staging Restaging	45/50 (90) 5/50 (10)
Subtype of Marginal Zone Lymphoma	Nodal	27/50 (54)
	Extranodal	21/50 (42)
	Splenic	2/50 (4)
Therapies prior to scan	Chemotherapy Radiation Therapy Surgery	1/50 (2) 1/50 (2) 3/50 (6)

## CXCR4-targeted PET/CT

Siemens Biograph mCT (64 or 128; Siemens Medical Solution, Erlangen, Germany) was used to conduct PET/CT scans from the vertex to mid thighs approximately 60 min after administration of [^68^ Ga]Ga-PentixaFor. Further details on PET-based reconstruction, applied CT protocols and radiotracer preparation are given in [2].

## Scan Interpretation

For interpreting scans, access to a workstation (syngo.via, VB50; Siemens Healthineers, Erlangen, Germany) was granted to four readers with varying levels of experience reading [^68^ Ga]Ga-PentixaFor PET/CT in lymphoma patients. Experienced observer was defined as more than four years of experience in interpretation of CXCR4-directed PET/CTs, and less experienced observer were defined as less than two years of experience in scan interpretation. All observers had no clinical information except for data provided in Table 1.

The following 8 diagnostic scan parameters were recorded by each reader: overall scan result, CXCR4 density in lymphoma tissue, extranodal organ involvement, No. of affected extranodal organs and extranodal organ metastases, lymph node (LN) involvement, No. of affected LN areas and LN metastases. We applied a binary assessment for overall scan result, extranodal organ and LN involvement and a six-point scale for all remaining (no.-related) items.

## Statistics

As described by Cicchetti [13], intraclass correlation coefficients (ICC) were used, which are based on a four-point scale to classify agreement rates. In brief, concordance is poor if the ICC is less than 0.4, fair between 0.4 to 0.59, good concordance ranges from 0.6 to 0.74, while excellent agreement rates achieve a minimum ICC of 0.75. We also provided 95% confidence intervals (CI). To compare performance among all readers, we also calculated the ICC for varying reader subgroups (experienced vs less experienced readers). Additional evaluation of Cronbach´s alpha as a reliability test was also performed [14]. Values larger 0.7 are considered as acceptable, values between 0.81 and 0.9 as good and larger 0.9 as excellent [15]. Cohen’s Kappa as reliability test within the group of the experienced readers and the group of the less experienced readers was also assessed [16]. Values between 0.41 and 0.60 are considered as moderate, between 0.61–0.80 as good and larger 0.80 as very good [17]. P value of 0.05 or lower was considered statistically significant. MedCalc statistical software (version 22.0.13; MedCalc Software bvba, Ostend, Belgium) was applied.

## Results

### Mainly Good to Fair Interobserver Agreement Rates

**Fig. 1.**
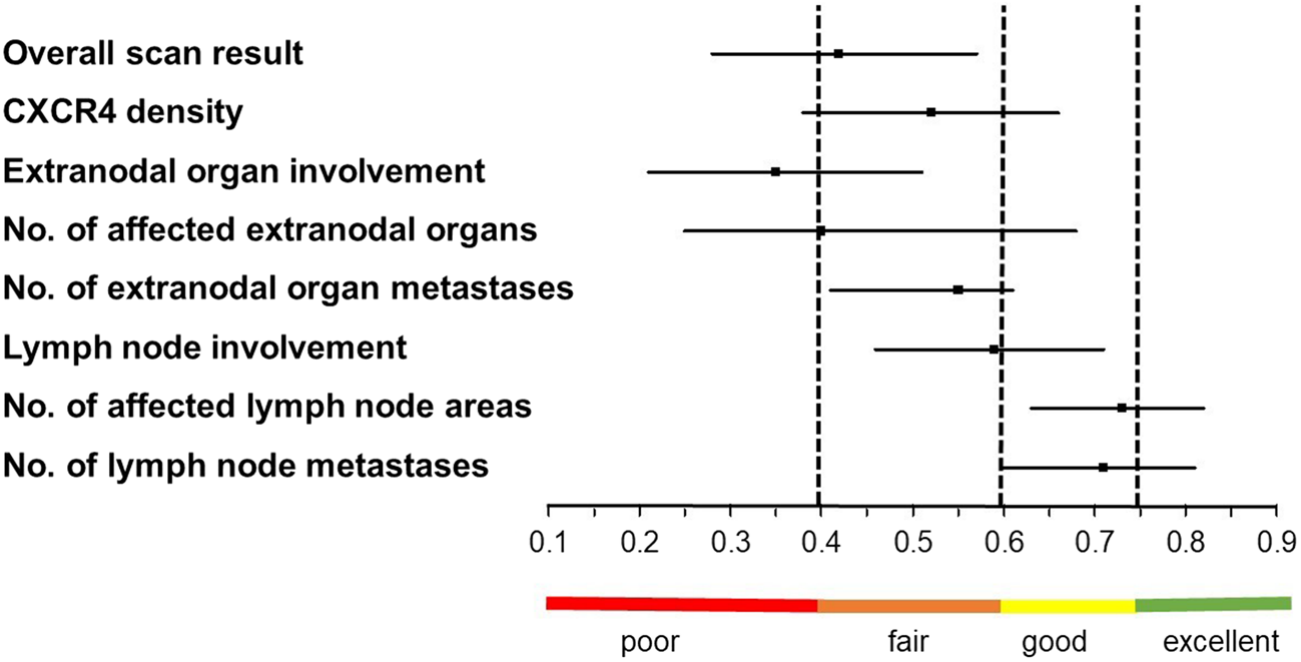
Forest plot for diagnostic scan parameters based on CXCR4-directed PET/CT. Extranodal parameters (organ involvement and number [No.] of affected organs) achieved poor concordance. The remaining parameters, however, achieved fair to good intraclass correlation coefficients (ICC).

Among all readers, the following agreement rates were recorded for diagnostic scan parameters (ranging from highest to lowest ICC): Good concordance was seen for No. of affected LN areas (ICC, 0.73; 95%CI, 0.63–0.82) and No. of LN metastases (ICC, 0.71; 95%CI, 0.60–0.81). Fair concordance was achieved for LN involvement (ICC, 0.59; 95%CI, 0.46–0.71), CXCR4 density in lymphoma tissue (ICC, 0.52; 95%CI, 0.38–0.66), No. of extranodal organ metastases (ICC, 0.55; 95%CI, 0.41–0.61), overall scan result (ICC, 0.42; 95%CI, 0.28–0.57) and No. of affected extranodal organs (ICC, 0.40; 95%CI, 0.25–0.68). Only extranodal organ involvement (ICC, 0.35; 95%CI, 0.21–0.51), however, achieved only poor concordance (Table 2). Figure 1 provides an overview of all diagnostic scan parameters. Additional evaluation of Cronbach’s alpha revealed comparable results with 7/8 (87.5%) of the investigated parameters achieving at least acceptable agreement rates (Supplementary Table 1).

**Table 2:** Overview of intraclass correlation coefficients (ICC) for assessment of diagnostic scan parameters. 95% confidence intervals are given in brackets

Parameter	ICC
Overall scan result	0.42 (0.28–0.57)
CXCR4 density in tumor tissue	0.52 (0.38–0.66)
Extranodal organ involvement	0.35 (0.21–0.51)
No. of affected extranodal organs	0.40 (0.25–0.68)
No. of extranodal organ metastases	0.55 (0.41–0.61)
LN involvement	0.59 (0.46–0.71)
No. of affected LN areas	0.73 (0.63–0.82)
No. of LN metastases	0.71 (0.60–0.81)

No. = number. LN = lymph node

**Fig. 2.**
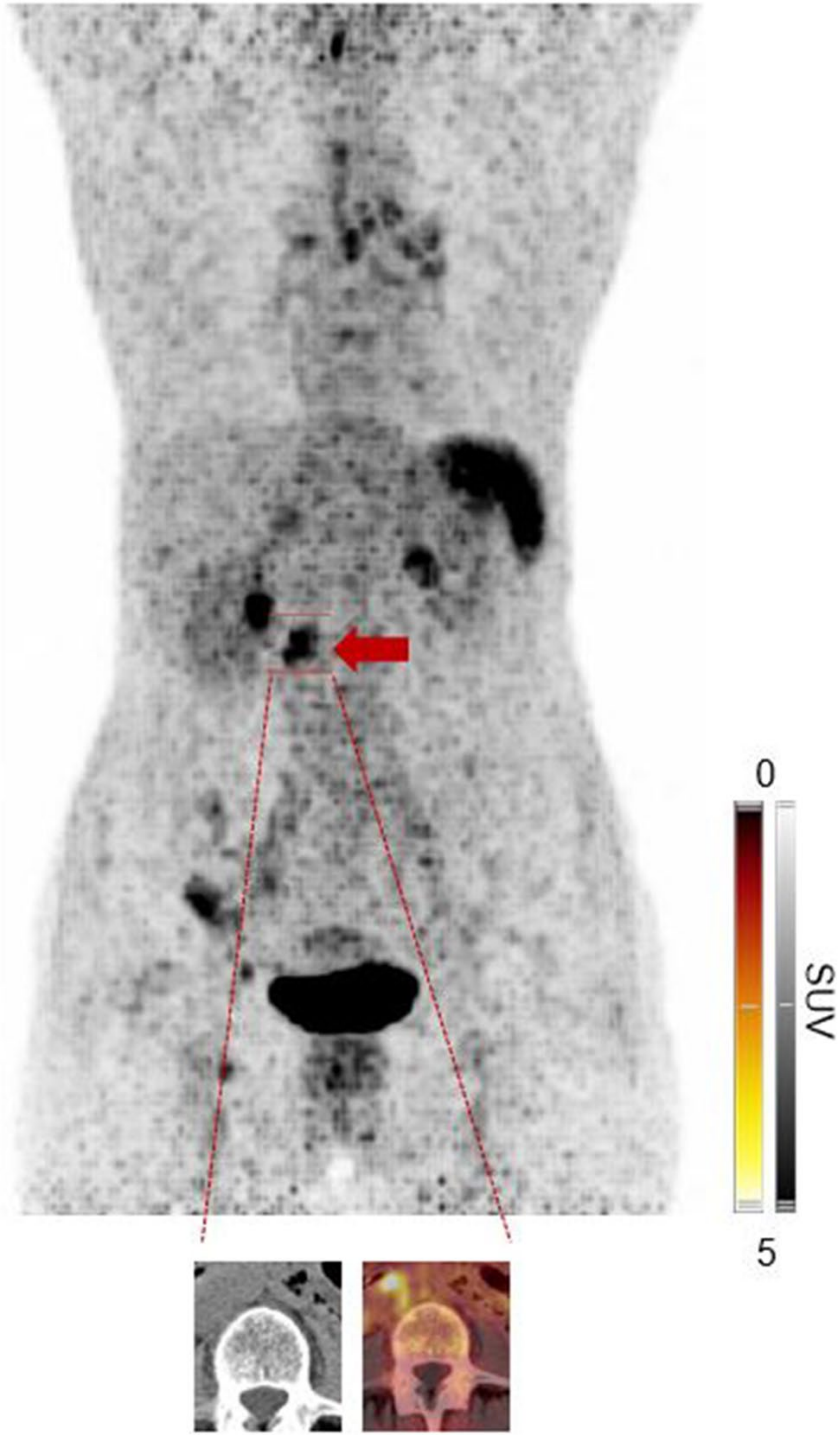
66-year old subject diagnosed with nodal marginal zone lymphoma. As seen on the maximum intensity projection, there were nodal lymphoma manifestations above and below the diaphragm, along with a CXCR4-positive lymphoma manifestation in the intestine (transaxial CT and PET/CT on the bottom). This extranodal involvement was only identified by the experienced, but not by the less experienced readers, highlighting the challenge of interpreting manifestations outside of the nodal system using CXCR4-targeted PET/CT.

Figure 2 displays a patient diagnosed with nodal MZL. In addition to nodal lymphoma manifestations above and below the diaphragm, there is a CXCR4-positive lymphoma manifestation in the intestine. This extranodal involvement was detected by the experienced, but not by the less experienced readers, highlighting the challenge of interpreting manifestations outside of the nodal system.

## High Level of Experience is Required for Scan Interpretation

When compared to less experienced observers, experienced readers achieved the highest significant agreement rates in 4/8 (50.0%) investigated scan items (ICC, range, 0.21–0.90, P < / = 0.04). In the remaining 4/8 (50%) of the parameters, a similar trend with higher ICCs for the experienced readers was recorded. Those findings may indicate that a high level of reader experience is required to interpret CXCR4-directed PET/CT in lymphoma patients (Table 3). Additional Cohen’s kappa evaluation provided comparable results with significant values for experienced readers when compared to less trained interpreters (Supplementary Table 2).

**Table 3:** Comparison of reader’s level of experience. Intraclass correlation coefficients (ICC) for all investigated parameters are indicated for the two experienced readers (left column), and the two less experienced readers (middle column). 95% confidence intervals are given in brackets. Per item, highest significant ICCs among the two subgroups are highlighted in bold and italic, thereby showing that the highest significant agreement rates were achieved in 4/8 (50%) instances for the experienced group (left). In the remaining 4/8 (50%) of the parameters, a similar trend was noted with higher ICCs for the experienced readers, indicating that there may be linear, experience-based relation when reading CXCR4-PET/CTs in MZL

Parameter	ICC Exp	ICC Less exp	Significance of difference
Overall scan result	0.46 (0.21–0.65)	0.30 (0.02–0.53)	P = 0.36
CXCR4 density in lymphoma tissue	0.61 (0.40–0.76)	0.50 (0.22–0.68)	P = 0.44
Extranodal organ involvement	***0.46 (0.21–0.65)***	0.03 (0.01–0.30)	**P = 0.02**
No. of affected extranodal organs	***0.48 (0.23–0.67)***	0.13 (0.00–0.40)	**P = 0.04**
No. of extranodal organ metastases	0.65 (0.46–0.79)	0.41 (0.15–0.61)	P = 0.10
LN involvement	0.75 (0.60–0.85)	0.53 (0.30–0.70)	P = 0.06
No. of affected LN areas	***0.86 (0.77–0.92)***	0.63 (0.43–0.77)	**P = 0.01**
No. of LN metastases	***0.82 (0.71–0.90)***	0.61 (0.41–0.76)	**P = 0.03**

No. = number. Exp = experienced, Less exp = less experienced

## Discussion

In the present study investigating levels of agreement on CXCR4-targeted PET/CT in patients with MZL, we observed a fair to good concordance rate for almost all diagnostic scan parameters, except for extranodal organ involvement. When comparing ICCs among the subgroups based on their level of experience in interpreting [^68^ Ga]Ga-PentixaFor PET/CT, the highest concordance was observed in the experienced group, thereby indicating that training may be needed to properly interpret such scans. Of note, as additional analyses, we performed Cronbach´s alpha for interrater reliability for the four raters and Cohen´s Kappa for the comparison of the group of experienced readers vs less experienced readers. Again, we observed comparable results with lowest Cronbach’s alpha for extranodal organ involvement and significantly higher Cohen’s kappa for experienced readers.

CXCR4-directed imaging and therapy has been extensively used in recent years (2–6) and may be most appropriate in patients with hematological malignancies [5]. Among those patients, MZL appears to be one of the most promising subgroups for chemokine receptor-directed imaging [2], as this lymphoma subtype provided high CXCR4 levels on immunohistochemical assessment [1] and substantially high radiotracer accumulation in patients imaged with CXCR4-targeted [^68^ Ga]Ga-PentixaFor [5]. Of note, recent studies involving up to 100 MZL patients also provided evidence on the high impact of this PET agent on the diagnostic algorithm [2]. However, prior to a more widespread use, interobserver rates should be investigated, as such an approach will then ensure that multiple readers, preferably from different imaging centers, arrive to identical (or at least comparable) results in scan interpretation [8]. We observed fair to good concordance for seven out of eight diagnostic scan parameters, while the remaining item is referred to extranodal disease (Fig. 1). The low agreement for extranodal organ involvement can rather not be explained by the patient population, as extranodal and nodal subtypes were balanced in our cohort (Table 1). Duell et al. recently reported on varying extranodal manifestations on CXCR4-directed PET/CT in MZL, including gastrointestinal or splenic region in up to 18% of the patients [2]. Those compartments, however, are also part of the biodistribution of [^68^ Ga]Ga-PentixaFor PET [18], which makes it challenging to identify sites of lymphoma involvement in those organs. Those considerations are further fueled by the fact that extranodal parameters also did not consistently achieve increased ICC in the subgroups with less experienced readers (Table 3). Taken together, in line with previous results investigating the rate of up-/downstaging when compared to guideline-compatible assessment [2, 7], CXCR4-directed PET/CT appears promising for staging purposes, which, however, seems to be dependent of previous level of experience. In this regard, we also observed that relative to less trained interpreters, experienced readers achieved higher ICC values in half of the investigated scan items, with a similar trend in the remaining parameters. This was also seen for additional statistical tests (Cohen’s kappa, Supplementary Table 2). As such, further studies should investigate such a potential linear trend between experience and scan interpretation, preferably by including more trained reading experts. Nonetheless, given the fact that previous reports also indicated a link between signal strength on CXCR4-directed PET/CT and progression-free survival [2], the herein observed concordance on diagnostic scan parameters may pave the way for [^68^ Ga]Ga-PentixaFor PET in (interim) response assessment for MZL, e.g., by applying novel therapies [19]. In this regard, agreement rates should be further increased and structured reporting systems that have already been introduced for other theranostic agents may help to address this challenging task [20–22]. Future efforts such a revised version of the harmonization project in lymphoma may also include reporting criteria for CXCR4-directed PET/CTs [23].

The present study has limitations, including its retrospective nature and limited number of investigated patients. Future studies should address those aspects, e.g., in the prospective LYMFOR trial (EU CT No 2022–500918-25) focusing on CXCR4-targeted PET/CT in MZL among different readers and centers [24].

## Conclusion

CXCR4-directed PET/CT mainly provided fair to good agreement rates for scan assessment, while a high level of experience seems to be required for an accurate imaging read-out. Future efforts may turn towards interpretative scan harmonization or standardized reporting for chemokine receptor-directed molecular imaging in lymphoma patients.

## Supplementary Information

Below is the link to the electronic supplementary material.

Supplementary file1 (DOCX 21 KB)

## Data Availability

The main data presented in this study are available in the article. Detailed information are available on reasonable request from the corresponding. author.
