# Supplementary material for: Interobserver Agreement Rates on CXCR4-Directed PET/CT in Patients with Marginal Zone Lymphoma
Source: Mol Imaging Biol. 2024 Aug 1;26(5):774–9. doi: 10.1007/s11307-024-01940-y (PMC11436430; doi:10.1007/s11307-024-01940-y)
Supplement: Supplementary file 1 — Supplementary file1 (DOCX 21 KB) [file 11307_2024_1940_MOESM1_ESM.docx]

**SUPPLEMENTARY MATERIAL**

| Parameter | Cronbachs´s Alpha | 95% lower confidence interval |
| --- | --- | --- |
| Overall scan result (pos/neg) | 0.743 | 0.629 |
| CXCR4 density in tumor tissue | 0.815 | 0.733 |
| Extranodal organ involvement (yes/no) | 0.685 | 0.546 |
| No. of affected extranodal organs (0-5) | 0.725 | 0.603 |
| No. of extranodal organ metastases (0-5) | 0.827 | 0.751 |
| Lymph Node involvement (yes/no) | 0.851 | 0.785 |
| No. of affected LN areas (0-5) | 0.916 | 0.879 |
| No. of LN metastases (0-5) | 0.908 | 0.867 |

**Supplementary Table 1**. **Overview of Cronbach’s alpha for assessment of diagnostic scan parameters.** 95% confidence intervals are given in brackets. No.=number. LN=lymph node.

| Parameter | Cohen´s Kappa Exp | Cohen´s Kappa Less exp | Significance of difference |
| --- | --- | --- | --- |
| Overall scan result (pos/neg) | 0.45 (0.12-0.79) | 0.29 (-0.09-0.67) | P = 0.37 |
| CXCR4 density in tumor tissue | 0.40 (0.19-0.61) | 0.43 (0.21-0.65) | P = 0.86 |
| Extranodal organ involvement (yes/no) | ***0.45 (0.18-0.72***) | 0.02 (-0.23-0.28) | **P = 0.02** |
| No. of affected extranodal organs (0-5) | 0.42 (0.20-0.63) | 0.06 (-0.13-0.26) | P = 0.06 |
| No. of extranodal organ metastases (0-5) | 0.55 (0.35-0.74) | 0.28 (0.05-0.51) | P = 0.11 |
| Lymph Node involvement (yes/no) | ***0.75 (0.57-0.94)*** | 0.51 (0.26-0.77) | **P = 0.04** |
| No. of affected LN areas (0-5) | 0.73 (0.60-0.85) | 0.51 (0.34-0.68) | P = 0.07 |
| No. of LN metastases (0-5) | ***0.76 (0.62-0.90)*** | 0.53 (0.34-0.72) | **P = 0.05** |

**Supplementary Table 2. Comparison of reader’s level of experience. Cohen’s kappa for all investigated parameters is indicated for the two experienced readers (left column), and the two less experienced readers (middle column).** 95% confidence intervals are given in brackets. Per item, highest significant ICCs among the two subgroups are highlighted in bold and italic, thereby showing that the highest significant agreement rates were achieved in 3/8 (37.5%) instances for the experienced group (left). In the remaining 5/8 (62.5%) of the parameters, a similar trend was noted with higher ICCs for the experienced readers, indicating that there may be linear, experience-based relation when reading CXCR4-PET/CTs in MZL. No.=number. Exp=experienced, Less exp= less experienced.
